# Monogenic disorders as mimics of juvenile idiopathic arthritis

**DOI:** 10.1186/s12969-022-00700-y

**Published:** 2022-06-18

**Authors:** Laura Furness, Phil Riley, Neville Wright, Siddharth Banka, Stephen Eyre, Adam Jackson, Tracy A. Briggs

**Affiliations:** 1 Royal Manchester Childrens Hospital, Manchester University NHS Foundation Trust, Manchester, UK; 2 Department of Paediatric Rheumatology, Royal Manchester Childrens Hospital, Manchester University NHS Foundation Trust, Manchester, UK; 3 NW Genomic Laboratory Hub, Manchester Centre for Genomic Medicine, St Mary’s Hospital, Manchester University NHS Foundation Trust, Manchester, UK; 4 Division of Evolution and Genomic Sciences, School of Biological Sciences, University of Manchester, Manchester, UK; 5 The University of Manchester, Versus Arthritis Centre for Genetics and Genomics, Centre for Musculoskeletal Research, Manchester, UK; 6 Manchester Academic Health Science Centre, NIHR Manchester Biomedical Research Centre, Manchester University NHS Foundation Trust, Manchester, UK; 7 Manchester Centre for Genomic Medicine, Division of Evolution & Genomic Sciences, School of Biological Sciences, Faculty of Biology, Medicine and Health, University of Manchester, Manchester, M13 9WL UK

**Keywords:** Juvenile idiopathic arthritis, Mimics, Multicentric carpotarsal osteolysis syndrome, Camptodactyly-arthropathy-coxa vara-pericarditis syndrome, Blau syndrome, Monogenic, Genetic syndromes

## Abstract

**Background:**

Juvenile idiopathic arthritis is the most common chronic rheumatic disease of childhood. The term JIA encompasses a heterogenous group of diseases. The variability in phenotype of patients affected by the disease means it is not uncommon for mimics of JIA to be misdiagnosed.

**Case presentation:**

We present four cases who were treated in single tertiary rheumatology centre for JIA who were subsequently diagnosed with a rare monogenic disease. All four patients shared the unifying features of presenting in early childhood and subsequently suffered with refractory disease, not amenable to usual standards of treatment. Multicentric Carpotarsal Osteolysis Syndrome and Camptodactyly-arthropathy-coxa vara-pericarditis syndrome are non-inflammatory conditions and patients typically present with arthropathy, normal inflammatory markers and atypical radiological features. Blau syndrome is an autosomal dominant condition and patients will typically have symmetrical joint involvement with a strong family history of arthritis, signifying the genetic aetiology.

**Conclusions:**

We share our learning from these cases to add to the growing portfolio of JIA mimics and to highlight when to consider an alternative diagnosis. In cases of refractory disease and diagnostic uncertainty further imaging and genetic testing can play a crucial role in establishing the aetiology. In all of these cases the correct diagnosis was made due to careful, longitudinal clinical phenotyping and a close working relationship between rheumatology, radiology and clinical genetics; highlighting the importance of the multidisciplinary team in managing complex patients.

## Background

Juvenile idiopathic arthritis (JIA) is a heterogenous group of diseases characterized by arthritis of unknown origin with onset before age of 16 years [1]. It is the most common chronic rheumatic disease of childhood. JIA is the umbrella term for all arthritides lasting longer than 6 weeks and diagnosis requires recognition of distinctive patterns of disease to differentiate rheumatic causes of arthritis from non-rheumatological causes [2]. A diagnosis of JIA has a significant impact on a child and their family. It imposes a significant burden often involving complex management plans with multiple hospital visits, pharmacological interventions, physiotherapy, occupational therapy and psychosocial impact. This can interfere with school attendance, social interactions and family life, as well as having a significant financial cost to the health service [3].

There is no single diagnostic investigation for JIA and the aetiology is complex. Using genome wide association studies, we have previously identified 14 new loci and validated three previous loci associated with JIA [4]. Such large-scale genetic studies have allowed critical pathways in disease pathogenesis to be highlighted, but often do not provide insight into aetiology for individual patients.

An alternative genetic approach, which can be utilised to interrogate the genetic drivers of autoimmune disease, is through the study of rare, familial monogenic forms of JIA or JIA like disease. Examples of such studies include the identification of *LACC1* biallelic mutations, such as described in 13 patients with systemic JIA from 5 consanguineous families [5] and the diagnosis of JIA in association with biallelic *ACP5* mutations [6]. This genetic approach may also be of value in the rheumatology clinic in cases of diagnostic uncertainty or refractory disease.

We present three distinct genetic syndromes in children who were misdiagnosed as JIA. All of these patients presented as JIA mimics with joint pain or deformity in early childhood. The long diagnostic odyssey experienced by these patients highlights the need to continue sharing cases of rare monogenic JIA mimics to raise awareness of unifying features of these syndromes and prompt clinicians to consider genetic testing and an alternate diagnosis.

## Case 1

**Fig. 1:**
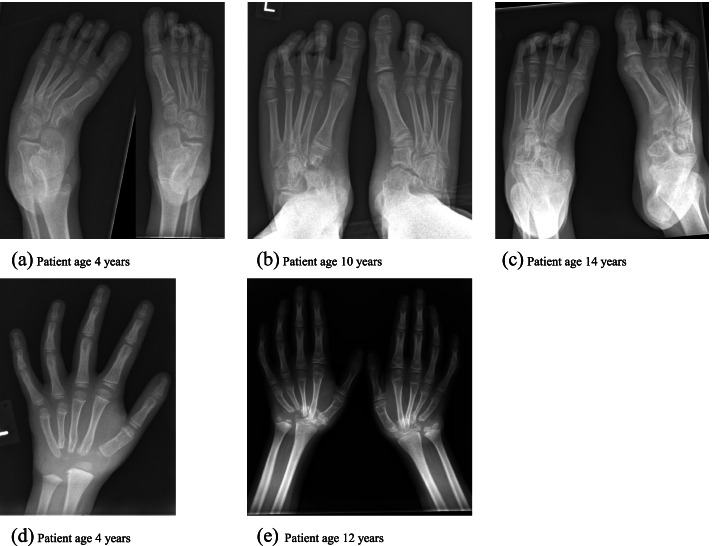
**a** – **c** Bilateral progressive erosive change and destruction primarily affecting the navicular and cuneiform bones over 10 years, aged 4 to 14 years. 1 (**d** – **e**) Bilateral destructive changes involving the carpal bones with subsequent foreshortening of the wrists and overlapping of the metacarpals over the distal radius

We describe a Caucasian male, born to non-consanguineous parents who presented as an infant with joint pain and swelling, particularly affecting the wrists, knees, ankles and feet. He was diagnosed with Rheumatoid factor (−), CCP (−) polyarticular JIA. There was no significant family history of rheumatological disease. When seen for the first time in the UK, age 4 years, he was on dual therapy of methotrexate and adalimumab. At this time, he was noted to have an abnormal foot position with a high arch and minimal movement of the midfoot. Initial inflammatory markers demonstrated a CRP <  1 mg/L and ESR 20 mm/1st hour. X-rays of the feet showed bilateral erosive changes, involving the tarsal and navicular bones with marked cavovarus (Fig. 1a).

Over the following 6 years he experienced ongoing foot stiffness, deformity and discomfort and had several courses of steroid injections into the talonavicular joint, which was noted to give some relief for short periods of time. At the age of 10 years, due to ongoing complaints of discomfort he was treated with a pulse of methylprednisolone and by age 11 years, etanercept was introduced. He was treated with one biologic and methotrexate. Despite significant navicular destruction noted on x-rays his distal phalanges and metatarsals were spared (Fig. 1b and c), he never mounted a significant inflammatory response biochemically, (CRP 1–6 mg/L, ESR 2-6 mm/1st hour). However, with a concurrent viral illness he did on one occasion have raised inflammatory markers of CRP 23 mg/L, ESR 40 mm/1st hour.

At age 12 years he was started on tocilizumab due to a disease flare; presenting with significant pain and restriction in both wrists. Serial x-rays demonstrated destructive changes of the carpal bone and proximal portions of the metacarpals (Fig. 1d & e).

**Fig. 2:**
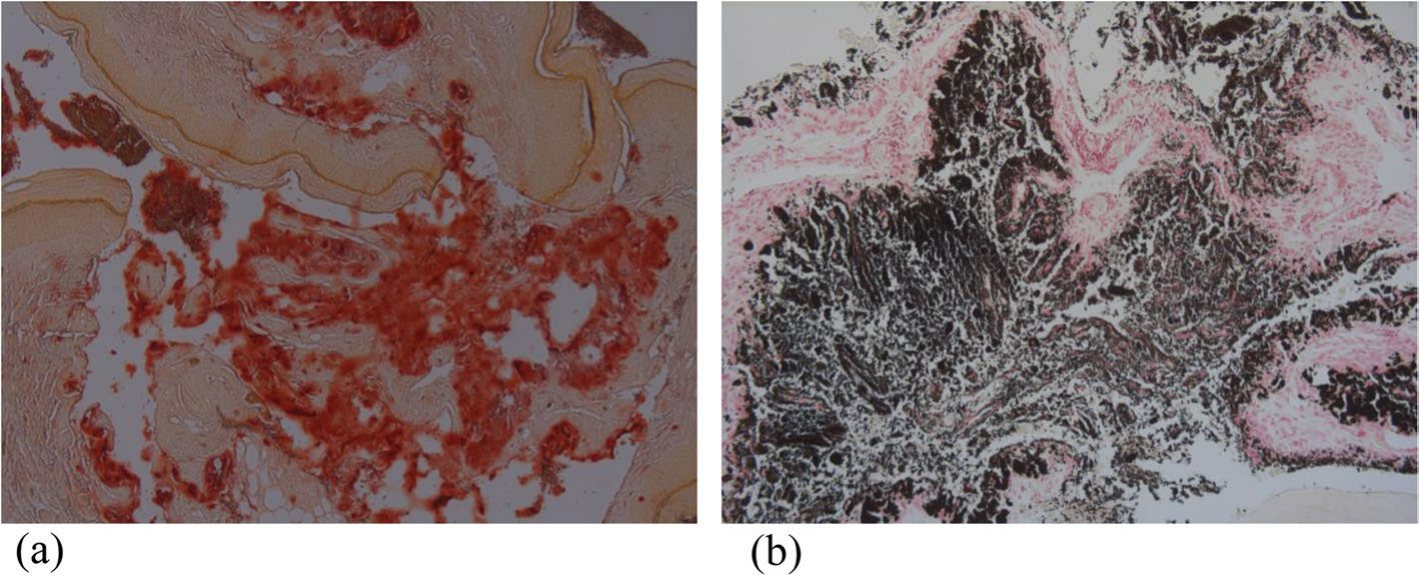
**a** Alizarin red staining positively for calcium units of calcium pyrosphate crystals in red 2 (**b**) Von kossa stains showing phosphate units of calcium pyrophosphate crystals in black

Ophthalmology review identified corneal clouding and a unilateral cataract. He received a formal diagnosis of Asperger syndrome at age 9 years. He had no obvious facial dysmorphism. At age 13 years he developed an erythematous soft tissue skin lesion on the right finger distal to the MCP, which was tender to touch. This was found to be benign, histology confirmed it contained both calcium and phosphate (Fig. 2a & b).

Given the x-ray findings in both hands and feet, Multicentric carpotarsal osteolysis (MCTO) was suspected clinically. This was confirmed through Sanger sequencing which identified a de novo heterozygous pathogenic variant in *MAFB* (c.176C > T, pPro59Leu) (Table 1). This variant, within the transactivation domain of the protein has been previously described in patients with MCTO [7, 8] and is thus considered consistent with a diagnosis of MCTO.

**Table 1:** Comparison of the major clinical features of the cases

Case	Condition	Characteristics	Present in our patient	Diagnostic delay (years)	Inheritance	History of consanguinity	Gene and variant description	Inflammatory markers	Imaging
1	MCTO	Osteolysis of Carpo-tarsal bones	+	12 years	AD	No	MAFB c.176C > T; pPro59Leu	CRP 1 – 6 mg/L ESR 2-6 mm/1st	x-ray
		Renal involvement	–					hour^a^	bilateral dissolution of bones in hands and feet
		Corneal clouding	–						
		Craniofacial abnormalities	–						
		Other manifestations (i.e skin deposit)	+						
2	CACP	Camptodactly	+	7	AR	Yes	PRG4 c.3462_3465delGACT;p.Thr1155LeufsTer7	ESR 1 -25 mm/1st hour	x-ray bilateral coxa vara with shallow acetabular and periarticular osteopenia^b^ US bilateral effusions of hips and knees MRI short and broad femoral necks with minimal enhancement with ring pattern and synovial thickening
		Arthropathy	+						
		Coxa vara	+						
		Pericarditis	+						
3	CACP	Camptodactly	–	12	AR	Yes	PRG4 c.2998_3001delAAAC;p.Lys1000LeufsTer43	ESR 1 – 9 mm/1st hour	
		Arthropathy	+						
		Coxa vara	+						
		Pericarditis	–						
4	Blau syndrome	Granulomatous		Unknown	AD	No	CARD15 (NOD2) c.1001G > A;p.Arg334Gln	Typically raised	No imaging available for this case
		Arthritis	+						
		Uveitis	Unknown						
		Dermatitis	Unknown						

^a^ESR noted to be 40 mm/1st hour on one occasion with intercurrent viral illness

^b^Osteopenia noted to be less severe than what is expected in JIA by reporting radiologist

MCTO is caused by heterozygous mutations in the MAF bZIP transcription factor B (*MAFB*) gene. MAFB is known to play a critical role in regulation of osteoclastogenesis and in normal renal development. In keeping with this role, the hall mark of MCTO is a skeletal dysplasia resulting in demineralization and osteolysis, mainly affecting the carpal and tarsal bones, as was observed in our case. In more than 50% of cases progressive nephropathy occurs leading to chronic renal failure [8]. Whilst our proband shows no signs of renal disease, we note that this has been reported across all ages, therefore we will maintain close follow up.

MCTO is also associated with corneal clouding and craniofacial abnormalities [9]. We note therefore the corneal clouding in our case. The finding of a cataract may relate to steroid use, but we note that *MAFB* is a transcription factor expressed in the eye and thus a role in cataract development could be postulated, although has not been described before.

With regards to the soft tissue digital lesion, containing calcium and phosphate in our case, we note that there have been previous descriptions of thickened skin and nodules over the planter surface associated with MCTO, but no known cases involving deposits [9]. The significance of which is not yet fully understood. Nor is it currently clear whether the diagnosis of Asperger syndrome relates to the underlying genetic diagnosis, but we note the case report of Upadia et al. [10], describing a child with MCTO and learning difficulties. The MCTO diagnosis raises important treatment questions. Whilst immunotherapy has not been proven to halt disease progression, a reduction in pain has been described in a patient treated with tocilizimab by Nishikomori et al. [11]. In our patient, biological treatment has been stopped now for 18 months, the patient continues to suffer with non-severe pain, managed with simple analgesics, currently with no significant worsening of destructive symptoms.

## Case 2 and 3

Case 2 is that of female child of South Asian origin, born to consanguineous parents who was admitted at age 2 years with a 4-month history of knee pain, swelling and fever. Ultrasound of the hips and knees confirmed synovitis and effusion. Examination also revealed bilateral camptodactyly and abnormal heart sounds. An ECHO demonstrated a moderate size pericardial effusion. Initial bloods showed positive RNP, ANA and cardiolipin antibodies (titres unavailable and subsequently negative), ESR 25 mm/1st hour.

In view of persisting joint arthropathy over the following 6 months, affecting the elbow, knee, shoulders and hips, despite treatment with methotrexate and prednisolone, treatment was escalated to anti-IL6 therapy, with 2-weekly infusions of tocilizumab. She then developed neutropenia and recurrent infections requiring admission.

**Fig. 3:**
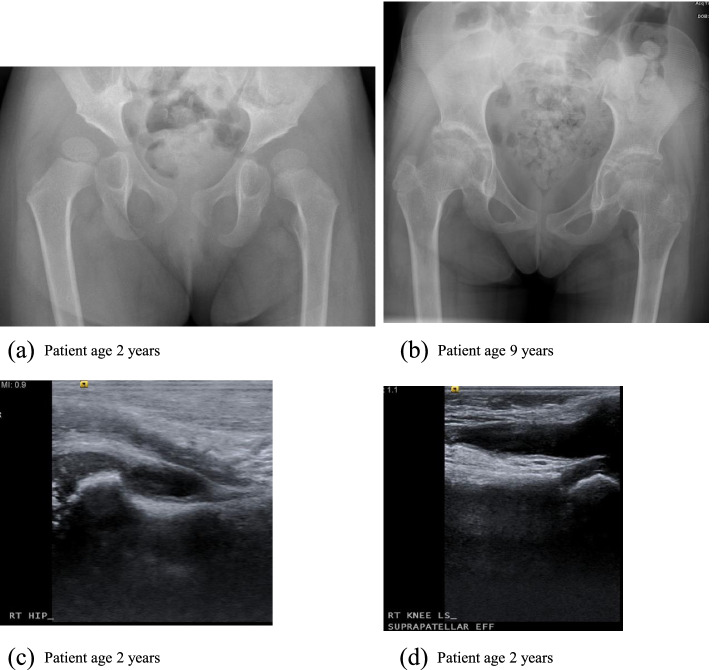
**a** x-ray pelvis showing mild bilateral acetabular dysplasia with a shallow acetabulum, (**b**) x-ray pelvis showing short and broad femoral neck with coxa vara and a minimal sclerosis of both acetabular roofs (**c**) US right hip shows a moderate effusion (d) US right knee shows a moderately large effusion in the suprapatellar pouch

Diagnosis was initially unclear and she underwent genetic testing for neonatal onset multisystem inflammatory disease (CINCA syndrome) which did not identify any pathogenic variants in *NLRP3*, furthermore histology of synovial fluid revealed a non-inflammatory picture, thus not consistent with CINCA. X-rays of the hips indicated mild acetabular dysplasia, with a shallow acetabular (Fig. 3a). Ultrasound confirmed bilateral hip and knee effusions (Fig. 3c & d). Further x-rays went on to demonstrate coxa vara, as demonstrated in Fig. 3b.

Given the combination of camptodactyly, non- inflammatory arthropathy and previous pericarditis a diagnosis of Camptodactyly-arthropathy-coxa vara-pericarditis syndrome (CACP) was considered and targeted exome sequencing revealed a homozygous *PRG4* pathogenic variant (c.3462_3465delGACT p.Thr1155LeufsTer7) (Table 1). Both parents were found to be heterozygous for the variant.

**Fig. 4:**
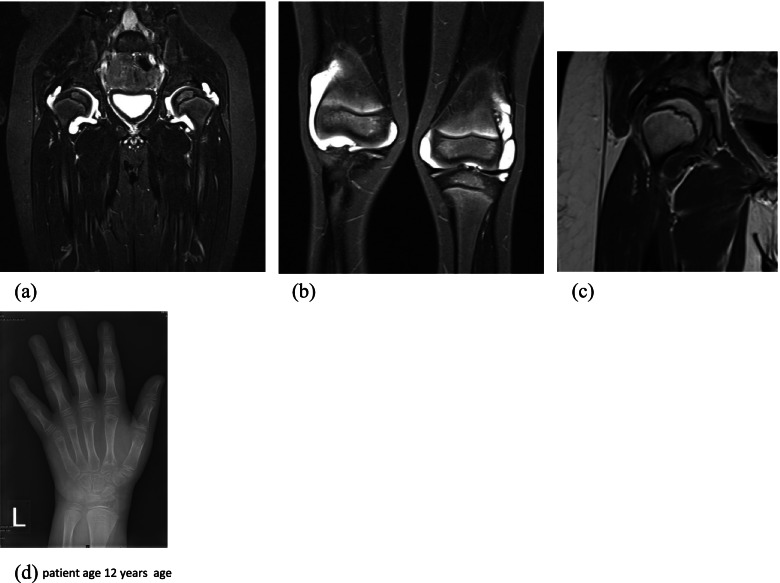
**a**– **b** b MRI STIR Coronal T2 sequences show symmetrical effusions affecting both hips a and knees joints, bilateral coxa-vara, with short and broad femoral necks, moderate effusions of the hips and knees (**c**) Coronal T1 post contrast shows a moderate effusion with minimal synovial enhancement (**d**) x-ray left wrist showing periarticular osteopenia

Case 3 was a male born to consanguineous parents; he presented in infancy with hypothyroidism and meningitis complicated by a subdural haematoma. At age 3 years he presented with joint swelling particularly affecting the elbows, knees and wrists and was noted to be hypermobile. X-rays revealed bilateral knee effusions; leading to an early diagnosis of Poly JIA. His treatment was escalated from methotrexate and joint injections to etanercept. He demonstrated mild developmental delay in speech and motor skills noted from infancy. On sequential MRI he was noted to have bilateral coxa-vara, with short and broad femoral necks, moderate effusions of the hips and knees with minimal enhancement and synovial thickening (Fig. 4 a, b & c). X-ray of the hands, age 12 years, demonstrated periarticular osteopenia (Fig. 4 d).

Immunological studies and autoantibody screen work up was normal. Given the history of learning difficulties, plus the above the patient had been recruited into the deciphering developmental disorders study (REF: PMID: 28135719), which did not initially identify a diagnosis. Additionally screening of 21 genes on the University College London next-generation sequencing autoinflammatory gene panel was undertaken and did not identify any pathogenic variants. A reanalysis of the family’s exome data from the DDD (#270616) at Manchester Centre for Genomic Medicine using a previously described pipeline (REF: PMID: 31637422; PMID: 30664714; PMID: 29276005) identified two homozygous frameshift mutations, one in *PRG4* (c.2998_3001delAAAC; p.(Lys1000LeufsTer43) (Table 1) and another in *TRHR* (c.745dupA; p.(Thr249AsnfsTer3)). His mother was found to be a heterozygous carrier of these variants, whilst testing was not possible in the father.

Camptodactyly arthropathy coxa vara pericarditis syndrome (CACP) is an autosomal recessive condition, caused by pathogenic variants in *PRG4* and characterized by the association of congenital or early onset camptodactyly, non-inflammatory arthropathy, progressive coxa vara deformity and/or pericardial effusion [12, 13]. The protein encoded by *PRG4*, lubricin, is synthesized at the surface of articular cartilage and present in synovial fluid; it functions as a boundary lubricant at the cartilage surface and inhibits synovial proliferation [13].

In our cases of CACP, we observed Camptodactyly in case 2 but not case 3. This was the first presenting feature in 68% of patients in a study of 35 cases reported previously [14]. CACP is typically symmetrical affecting large joints, with the wrists as the first joint affected in some case reports [15, 16]; importantly, synovial fluid analysis reveals non-inflammatory changes.

To differentiate CACP with imaging; typical features include, lack of erosive changes and periarticular osteopenia, as was seen in our cases. Although osteopenia is seen in JIA, a distinguishing feature of CACP is the squaring of metacarpals and phalanges [14, 17]. Large acetabular cysts on pelvic radiographs are not seen in JIA and are a feature of CACP, a feature which could be argued to be pathognomonic [17]. MRI enhancement of a joint capsule with a ring pattern is typical of CACP as opposed to a solid enhancement pattern seen in JIA. Coxa vara presents clinically with increasing age, which could contribute to the delay in early diagnosis of CACP [12] .

We noted pericarditis in case 2 but not case 3; this is a feature which has been reported in up to 30% of published cases from Yilmaz et al. [14].

A combination of lack of clinical signs of inflammation and careful examination of radiological features should prompt CACP as a differential diagnosis. Prognosis is progressive, with joint contractures worsening over time due to insufficient lubrication between tendon and tendon synovium, increasing hip pain is thought to reflect the accumulation of mechanical ware [14].

There is no current standard medical treatment for the arthralgia, anti-inflammatory medications provide little relief in this non-inflammatory condition. Treatment is focused on muscle strengthening and building. However, there has been a case report of total hip arthroplasty to relive pain and improve function in siblings with CACP. Both patients’ had the procedures electively and reported improvements in symptoms, joint replacement is not widely adopted into routine practice [18].

In both of our cases biologics were stopped when the diagnosis was confirmed; neither has experienced any worsening of symptoms or progression of disease over the past 14 months and one patient reports feeling better since stopping medication. With adolescent growth the knee contractures in patient 2 have improved but her elbow contractures remain severe.

It is believed PRG4 functions as an effective ocular surface boundary lubricant [19] and a case of CACP with bilateral cataracts has been previously reported [20].

## Case 4

In one Caucasian family the proband presented with an inflammatory arthritis resulting in the diagnosis of JIA in childhood and subsequently in adult life when had children, both of his sons were also diagnosed with JIA. Given the strong family history in this case whole exome sequencing was undertaken and revealed a heterozygous pathogenic variant in *CARD15* (*NOD2*) (c.1001G > A; p.Arg334Gln) in all three affected individuals, which was absent in the unaffected mother of both sons (Table 1). This variant was confirmed by Sanger sequencing.

Genetic variants in *NOD2* have previously been associated with Crohn’s disease, early onset Sarcoidosis and Blau syndrome [21]. Blau syndrome is a rare, autosomal dominant disorder characterized by the triad of granulomatous arthritis, uveitis, and dermatitis with age of onset typically in infancy [22]. The three family members we report manifested the clinical features of an inflammatory arthritis resulting in the diagnosis of JIA, whilst uveitis, skin lesions and camptodactyly were not reported. It is of note that all features of Blau syndrome are not required to make the diagnosis clinically and we are unable to review the family to assess for any further development of symptoms, although note that the proband was recruited to our study in adulthood.

The specific *NOD2* variant identified in our family has previously been described in association with Blau syndrome in four individuals from two families [23]. Both affected individuals in one of the families manifested all key features of Blau syndrome, whilst both individuals in the second family suffered from joint inflammation and one additionally manifested skin lesions and the other camptodactyly, but additional features were not reported.

The NOD2 protein has several critical functions including in recognising bacteria and stimulating the immune response, autophagy and apoptosis. Pathogenic variants in Blau syndrome have been shown to increase activity of the NF-kB signalling pathway, activating inflammatory genes [24].

## Discussion

JIA is the most common chronic childhood arthritis and is a diagnosis of exclusion. It is a heterogenous condition and there is no single diagnostic marker for JIA, making it difficult to distinguish the disease from mimics that present in a similar way. The patients described in this series demonstrate key clues clinicians can look out for to distinguish JIA from monogenetic mimics.

Multicentric carpotarsal osteolysis syndrome and Camptodactyly-arthropathy-coxa vara-pericarditis syndrome are non-inflammatory conditions. The patients described above in cases 1–3 did not mount significant inflammatory responses with ESR typically ranging from 1 to 9 mm/1st hour. Although patients may complain of arthralgia, joints are not swollen and warm to touch, as would be expected in JIA.

These conditions did not show a response to immune modulating treatments, which are effective in JIA such as high dose steroids, joint injections and biological therapies. The absence of raised inflammatory markers and the lack of response to numerous treatments should trigger consideration of an alternative diagnosis. With the significant advances in therapeutics to treat JIA, it is widely considered that remission of symptoms in the disease is achievable [25]. The treat to target model in paediatric rheumatology, essentially a model that advocates regular reassessments of disease activity, drug response and growth [26], ensures that when a patient fails to achieve these targets the treatment approach should be re-evaluated, including considering an alternative diagnosis. Failure to recognise an alternative diagnosis can lead to significant delays in diagnosis and poor outcomes for patients, as demonstrated in these cases with a delay in diagnosis ranging from 7 to 12 years.

Conventional radiology is well recognised as playing a pivotal role in assessing the extent and progression of joint involvement in JIA [27]. Typical features showing soft tissue swelling, loss of joint spaces and osteopenia. Patients presenting with atypical features or absence of these typical features on imaging should be considered for further investigation. The utility of different imaging modalities to differentiate JIA from JIA mimics is demonstrated in this case series, as shown in Table 2 [28–30]. For example, patients with progressive bone loss with subsequent skeletal deformities and functional impairment could have an inherited osteolysis disorder such as MCTO or nodulosis, arthropathy and osteoysis (NOA) syndrome. Ultrasound can be a can be a useful tool to differentiate JIA from a non-inflammatory process as seen in CACP as patients may have prominent synovial proliferation with normal synovial vascularity [31]. Although symmetrical joint involvement can be seen in JIA, additional image findings such as osteopenia with no erosions could direct you to a genetic disease such as Blau syndrome.

**Table 2:** Clinician, molecular and radiological comparison between JIA and JIA mimics to aid differentiation

Condition	Characteristics	Inheritance	Typical age of onset (years)	Typical inflammatory markers (normal or raised)	Physical examination	Typical Image findings
JIA	Arthritis lasting > 6 weeks	Multifactorial	Childhood (< 16)	Raised	Soft tissue swelling Stiffness (mainly morning) Warmth to touch Painful ROM of affected joints Small and large joints affected	x-ray findings - soft tissue swelling, loss of joint spaces, osteopenia, erosions, growth disturbances, joint subluxation (1) US findings – synovial proliferation, joint effusions MRI findings – synovitis, bone erosion, bone marrow oedema, enhancement (2)
MCTO	Osteolysis of Carpo-tarsal bones renal failure +/− Corneal clouding +/− Craniofacial abnormalities +/− Other manifestations ie skin changes +/−	AD	< 1	Normal	Deformity of hands and feet Stiffness and restriction in ROM in hands and feet	x-ray – progressive destruction of the carpal and tarsal bones
CACP	Camptodactly Arthropathy Coxa vara Pericarditis	AR	< 1	Normal	Fixed flexion deformity of the proximal interphalangeal joints (most commonly affecting the 5th digit) Limitation in ROM of the hips and knees	x-ray - bilateral coxa vara with shallow acetabular and periarticular osteopenia Large acetabular cysts US - bilateral effusions of large joints - hips and knees Prominent synovial proliferation with normal synovial vascularity MRI - short and broad femoral necks with minimal enhancement with ring pattern, synovial thickening
Blau syndrome	Granulomatous, arthritis, uveitis & dermatitis Visceral involvement +/−	AD	< 4	Raised	Painful ROM of affected joints Joint swelling Skin changes Ocular symptoms	x-ray - osteopenia, joint space narrowing with no erosions, typically symmetrical (3)

1. [28]

2. [29]

3. [30]

In addition, a confirmed genetic diagnosis allows targeted screening and monitoring. Our patients with MCTO and CACP needed ophthalmology screening. Patients with MCTO need close monitoring of renal function to detect nephropathy early [32] and in CACP patients one should have a low clinical index of suspicion for pericarditis.

Increased understanding of the disease mechanism may lead to targeted therapy. It is well known that the protein encoded by *PRG4*, Lubricin, in CACP functions as a boundary lubricant at the cartilage surface and inhibits synovial proliferation. Differential gene expression of lubricin has been found in the synovium of rheumatoid arthritis and osteoarthritis patients [33]. Lubricin is essential for long-term joint homeostasis [34] and its role as a potential novel recombinant therapy continues to be explored [35].

In MCTO, MAFB is known to play a crucial role in regulation of osteoclastogenesis and in normal renal development. Regev et al. have recently reported improvements in bone mineral density and stabilization of osteolysis in a patient with MCTO treated with denosumab [36]. Through increased understanding of the functional mechanism by which *MAFB* results in bone destruction, it is hoped that targeted therapies, possibly through rankL may be developed.

Identification of the genetic condition allows identification of other at-risk family members, for example CACP is an autosomal recessive condition, whilst MCTO and Blau Syndrome are autosomal dominant disorders. A genetic diagnosis can impact on a family’s reproductive choices due to risks to future pregnancies; families need to be appropriately supported and counselled on this.

In all of these cases described diagnosis was made due to careful, longitudinal clinical phenotyping and a close working relationship between rheumatology, genetics and radiology colleagues through multidisciplinary joint clinics and collaborative team work. By integrating patient and family care we were able to offer appropriate genetic testing and subsequent segregation studies.

This list of JIA mimics is not exhaustive and many other genetic diseases can present in similar ways such as H-syndrome, an autosomal recessive condition characterised by cutaneous hyperpigmentation and hypertrichosis as well as short stature and arthritis. Bloom et al. reported 5 patients identified by whole exome sequencing, who had all presented to paediatric rheumatologists prior to diagnosis [37].

There is currently no specific clinical guideline of when to consider genetic testing in patients with JIA. However, with the mainstreaming of genetic services and improved access to cheaper, advanced next-generation sequencing it feels like now, more than ever we need to be clear about when we should consider investigating patients for rare monogenic causes of JIA. We hope that by sharing these cases we can add to the growing portfolio of mimics of JIA to strengthen phenotyping, leading to earlier recognition of unifying features and recognition of when to consider targeted genetic testing.

## Conclusion / clinical lessons

These cases share the unifying features of early onset, unexpected lack of inflammatory response, failure to respond to treatments or achieve remission, atypical radiological features and a strong family history or consanguinity.

## Data Availability

Not applicable.

## Abbreviations

ACP5: Acid phosphatase 5, Tartrate resistant
ANA: Antinuclear antibodies
CACP: Camptodactyly arthropathy coxa vara pericarditis syndrome
CARD15 (NOD2): Nucleotide binding oligomerization domain containing 2
CINCA: Neonatal onset multisystem inflammatory disease
CRP: C-reactive protein
DDD: Deciphering developmental disorders study
ECHO: Echocardiogram
ESR: Erythrocyte sedimentation rate
JIA: Juvenile idiopathic arthritis
LACC1: Laccase (multicopper oxidoreductase) domain containing 1
MAFB: MAF bzip transcription factor B
MCP: Metacarpophalangeal joint
MCTO: Multi centric carpotarsal osteolysis syndrome
MDT: Multi-disciplinary team
MRI: Magnetic resonance imaging
NGS: Next generation sequencing
NLRP3: NLR Family pyrin domain containing 3
NF-kB: Nuclear factor-kB
PRG4: Proteoglycan 4
RNP: Ribonucleoprotein
UCL: University College London
